# The accreditation role of Councils on Chiropractic Education as part of the profession's journey from craft to allied health profession: a commentary

**DOI:** 10.1186/s12998-020-00329-2

**Published:** 2020-07-22

**Authors:** Stanley I. Innes, Charlotte Leboeuf-Yde, Bruce F. Walker

**Affiliations:** 1 College of Science, Health, Engineering and Education, Murdoch University, Murdoch, Australia; 2 Institute for Regional Health Research, University of Southern Denmark, DK-5000 Odense, Denmark

**Keywords:** Chiropractic, Accreditation, Profession, Council on chiropractic education

## Abstract

**Background:**

Chiropractors see themselves as well positioned to provide safe, effective and economical care for the on-going financial burden that spinal pain imposes. However, in many places of the world, the chiropractic profession continues to find itself struggling to gain acceptance as a mainstream allied health care provider. There is evidence of the existence of undesirable chiropractic practice patterns and it is in part due to some of the world’s accredited chiropractic programs. This indicated a need for scrutiny of international chiropractic educational accreditation standards, which are the responsibility of Councils on Chiropractic Education (CCEs). To this end we reviewed an emerging body of evidence about the chiropractic educational system in order to identify issues and make recommendations that may enhance professional acceptance through improved graduate outcomes and hopefully the quality of patient care. This commentary summarises the findings of that research.

**Main text:**

We reviewed recent relevant studies, including our own, into the role and function of CCEs and found that there is sufficient evidence to identify areas of concern that could be addressed, at least in part, by improvements to CCEs’ educational standards and processes. Areas included a lack of definitions for key terms such as, ‘chiropractic’, ‘diagnosis’, and ‘competency’, without which there can be no common understanding at a detailed level to inform graduate competencies and standards for a matching scope of practice. Further, there is some evidence to suggest that in some cases this level of detail is avoided in order to enable a “big tent” approach that allows for a diversity of approaches to clinical care to co-exist. This combined with the held view that chiropractic is “unique”, highly valued, and best understood by other chiropractors, explains how students and practitioners can cling to ‘traditional’ thinking. This has implications for public safety and patient quality of care.

**Conclusion:**

If chiropractic care is to gain mainstream acceptance worldwide then it needs to adopt, through revitalised CCE accreditation standards and processes, those of other allied healthcare professions and wholeheartedly embrace science, evidence-based practice and patient centred care.

## Background

It is well recognised that low back pain is placing an enormous financial burden on societies worldwide [1, 2]. Chiropractors are well positioned [3] to answer the call to action by eminent international researchers involved in the field [4, 5] to provide safe, effective and economical care [1, 6]. However, despite increasing numbers of chiropractors, total utilization rates internationally have not changed appreciably over the past 35 years [7]. This suggests that the profession is not gaining mainstream acceptance such that it is on equal grounds with other allied healthcare professions internationally [8].

For a profession to gain ‘respectability’ it should be located appropriately within the culture of society. This is partly evidenced by the existence of professional accredited schools and state licensing or registration for practitioners. By achieving legitimacy the profession may eventually be seen as an embodiment of knowledge and trustworthiness [9]. Obviously, there are many possible factors that could contribute to this complex issue, however one important component is that chiropractic educational programs should be of quality and demonstrate basic professional homogeneous standards [10].

For chiropractic the establishment of educational standards occurs via Councils on Chiropractic Education (CCEs). These CCEs are located in North America (CCE-USA) [11], Australia (CCE-A) [12], Canada (CCE-C) [13], and Europe (ECCE) [14]. There is also an international umbrella council of chiropractic education organization, the Chiropractic Council on Education International (CCE-International) [15].

These CCEs define the necessary knowledge, skills, attitudes, and competencies students should attain before graduating and entering practice [16, 17]. CCEs also detail and monitor, amongst other things, the required program content, facilities, faculty, and financial management of the chiropractic programs (CPs).

Evidence is available that CCEs’ standards and processes are not internationally homogeneous and this in part has contributed to concerns raised about chiropractic education [18–23]. This indicates a need for scrutiny of international chiropractic educational and practice standards detailed by CCEs to identify facets that could be changed to enhance the quality and hence the reputation of chiropractic education. The aspirational end result would be improved patient safety and quality of care.

It is acknowledged that there are a number of factors involved in changing CPs’ educational standards. These include matters involving CP staff, chiropractic registration and licensing boards, chiropractic associations, government departments, health consumer groups, and the public at large. Until recently there has been a paucity of research exploring chiropractic education standards, however recent studies have begun to critically review those making the standards (CCEs) [24, 25], chiropractic students who are engaged in the learning (as dictated by the standards) [23, 26, 27] and the standards themselves [28–31].

We are of the view that, at this time, the collective body of knowledge requires a synthesis of the existing information with the goal of making recommendations for positive change.

### Objectives

The objectives of this review were to summarize contemporary research about the role of CCEs and how this may be enhanced to facilitate improvement in the propriety of the chiropractic profession as an accepted mainstream allied healthcare provider, specifically, by examining;

1. Known issues confronting CCEs’ standards and processes
2. The differing regulatory environments of CCEs to find common ground
3. Insights of those from within CCEs
  We then follow with a conclusion and recommendations.

## Main text

### Examining known issues confronting CCE standards and processes

We believe there are several issues that have implications for the CCE standards and processes.

First, there is no longer an international organisation that encompasses all CCEs that can establish worldwide agreement on a homogeneous set of accreditation standards. This is because the CCE-USA withdrew from the CCE-International in 2016 [15], without a public statement as to why this has taken place. This makes it difficult for CCEs to meet and ‘harmonise’ their standards.

Secondly, there is mounting evidence of regional variations in standards. Chiropractic students from the United States of America attain significantly lower scores on their national graduation licencing (NBCE) exams when compared to those from Canadian CPs [32]. Some CCE experts have questioned the “quality” of the students being admitted to CPs [24] and suggested their entry to and graduation from CPs was motivated by financial concerns of their teaching institutions. This concern is not unique to chiropractic educational institutions [33, 34]. Also, experts worried about the possible public safety implications resulting from students unable to master the complexity and volume of information required to practice competently [25]. It is during the undergraduate years that the future chiropractor will assimilate the contents of the course. If the schools or students are substandard, this is very likely to have repercussions on the profession, and if this becomes common, it will impact on the general population.

Thirdly, chiropractors themselves express concerns about their levels of competence. Recently graduated European chiropractors felt underprepared for practice in competencies such as interprofessional collaboration, contributing to professional and scientific knowledge, practice managerial roles [35], and paediatric health issues [36]. Even when chiropractors are found to be confident in their ability, such as to practice in an evidence-based manner [37], the reality is that many do not use evidence to guide clinical decision making [38, 39]. This has consequences for health economics and the quality of patient care and their safety.

Fourthly, there is considerable diversity within the profession itself. The early formative thinking of chiropractic was centred on the manual correction of misalignments / lesions within the spine (*subluxations*) that interrupted the spinal nerves carrying the flow of *vital* forces. This interference of nerve supply was thought to be responsible for the cause of almost all disease [40]. Today, there is a division between this ‘traditional’ group (also known as ‘conservatives’, ‘fundamentalists’, or ‘unorthodox’) that subscribe to the concept that the spine is the centre of good health and the ‘evidence-friendly’ faction of the profession that focuses on musculoskeletal problems using a contemporary and evidence-based paradigm [41]. The larger the former group, the more difficult it will be for the chiropractic profession to become accepted as an allied health profession.

The ‘traditional’ or ‘conservative’ beliefs result in practitioners, who hold that a wide range of diseases and musculoskeletal complaints will be helped or prevented by removal of nerve interference [42, 43]. The most recent figures suggest that at least 20% of chiropractors have this exclusive vertebral subluxation focus, and even up to 70% believe this concept may be an important practice consideration [44]. This pattern of thinking was found to be more prevalent in graduates from certain CPs, suggesting that the regulation of chiropractic education may be a significant factor [19]. Clearly, the CCE in that specific region was accepting, or at least ignoring, such practice, whereas this may not be the case for other CCEs.

In conclusion, there is not a uniform face for chiropractic education. There is considerable diversity in the educational ‘philosophies’ adopted and openly disclosed by CPs. Diversity in educational delivery is not an inherently bad thing, however in the chiropractic context some CPs throughout the world, especially in the USA, still openly promote traditional values of *vitalist* thinking by continuing to promote the concept of *subluxations* [45, 46]. In contrast other CPs have taken the position that *vitalism* / *subluxation* has no place in an evidence-based curriculum and should only be taught in an historical context [47]. This diversity of views / beliefs is permitted to co-exist under the jurisdiction of the same accreditation agencies. We believe this continues to cause reputational damage for the broader profession.

### Examining differing regulatory environments of CCEs to find common ground

**Fig. 1:**
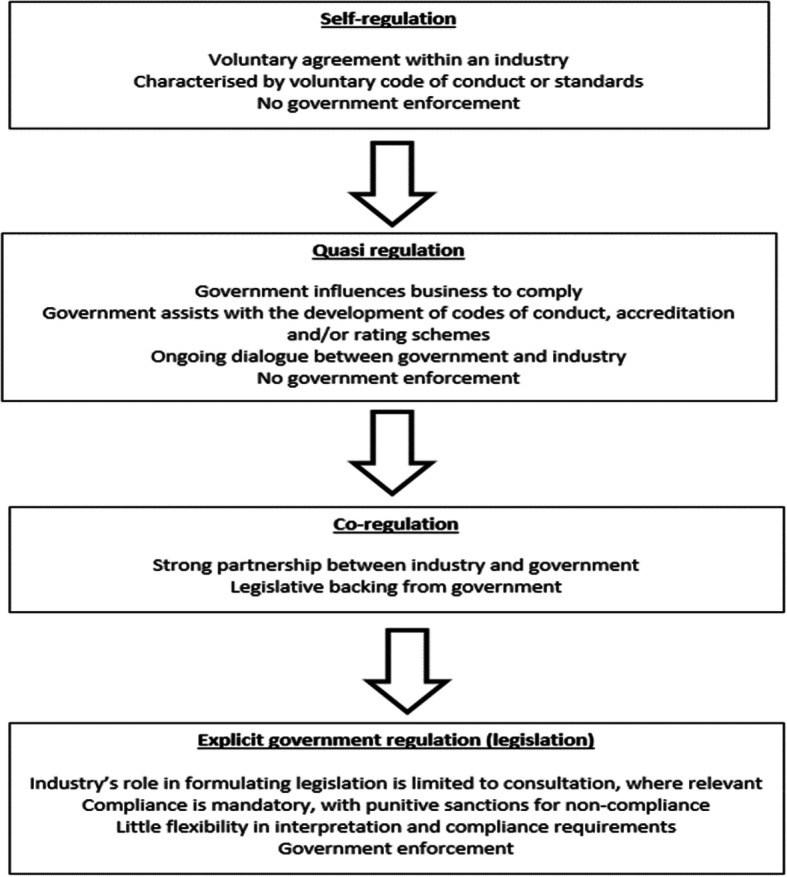
Explanation of regulation classification (Page 2, [49])

As previously discussed, not all CCEs belong to an international body, which works towards accepted minimum standards for chiropractic education. However, when asked, CCE experts did not believe it was possible for there to be an identical set of homogeneous international high-quality standards [25]. It was recognised that CCEs must function within their own regulatory and cultural environments and these are not uniform internationally. For example, the USA health education accreditation system is described as involving “quasi-regulation” [48] (Fig. 1). The CCE-USA is held to the standard by the USA Department of Education of showing itself to be a reliable authority regarding the quality of education of chiropractic programs [50]. Accordingly the CCE-USA describes its role as to “.. .*not define or support any specific philosophy regarding the principles and practice of chiropractic*. *.*. *.*. *(but) focus(es) on student learning outcomes that prepare graduates to serve as competent, caring, patient-centred and ethical doctors of chiropractic/chiropractic physicians qualified to provide independent, quality, patient-focused care to individuals of all ages*. .” [11]. The CCE-USA Council which consists of 18 members, 10 from chiropractic programs, 5 from private chiropractic practice and 3 public members, approves its own graduate competencies. This is done after consultation with “various stakeholders”, but without the requirement of government approval. Although likely considered there is no specific imperative for the protection of the public demanded by the U.S.A. Department of Education or the CCE-USA. As a consequence, there is no impediment for standards to be created that allow for chiropractic programs of various philosophies to gain accreditation that may have adverse consequences for patient care or potentially place the public at risk.

**Fig. 2:**
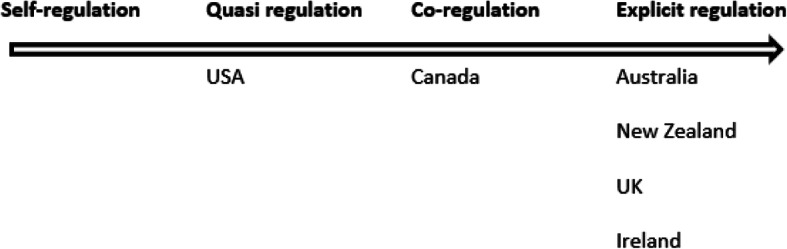
Classification of various regional health education accreditation systems (page 3, [49])

This sits in contrast to that of “co-regulation / explicit regulation” found in regions such as Australia (Fig. 2) (page 3, [45]) where there is a single National Registration and Accreditation Scheme that empowers and tasks government appointed National Boards with the primary directive to protect the public [41].

**Fig. 3:**
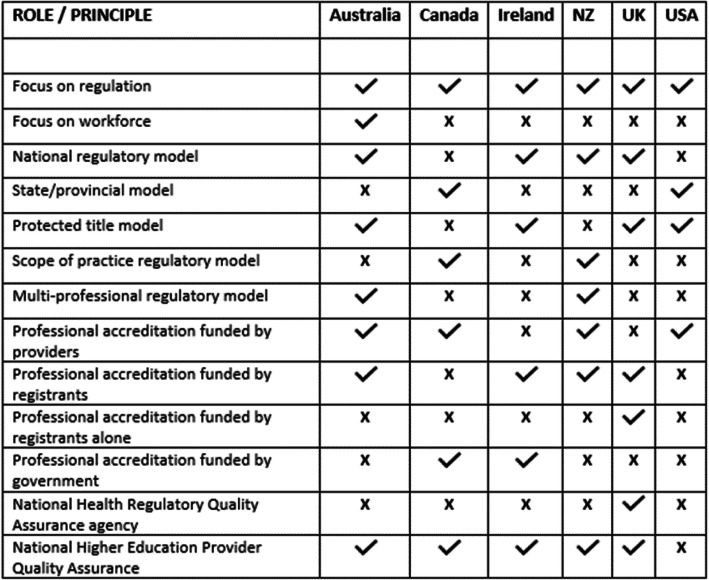
Comparison of roles of various regional health education accrediting authorities (page 5 [49].)

The detail of the differences between the regulatory differences between regions was the focus of a recent Australian Government review and a summary table is found in Fig. 3.

Thus, CCEs can be driven by differing primary objectives. Some may be focused on ensuring quality education while others are directed towards public safety. These are not mutually exclusive, and the nexus appears to be patient safety and quality of care. It is our opinion that the adoption of high-quality uniform CCE accreditation standards supported by the professional associations and regulatory bodies would align with other mainstream allied healthcare providers such as social workers, physiotherapists, and psychologists [49]. By doing so, the chiropractic profession could move further towards becoming an accepted and respected mainstream allied healthcare provider.

### Examining the insights of those within CCEs

A recent qualitative study seeking the views of key informants, who had extensive experience working within CCEs, offers insights that need to be considered when making recommendations for change [24, 25]. The picture that emerged was of a group of accreditation experts who perceived the chiropractic profession to be a collection of health care providers with diverse and strongly held views on the nature of chiropractic care [24]. The ‘conservative’ view of chiropractic was known to be vocal and determined to protect the historical roots of chiropractic care [51, 52]. The CCE experts valued the profession highly and perceived it to be special and generally best understood by chiropractors themselves. Finally, they saw themselves as having limited resources to carry out their functions.

Not surprisingly, the views of CCE experts were that accreditation standards were a result of compromises made to reconcile diverse and strongly held opinions about the scope of chiropractic practice in order to accommodate all practitioners’ perspectives [25]. This view is reinforced by the knowledge that subsequent revisions of standards have shown few signs of addressing undesirable consequential graduate practices and an unwillingness to adopt key standards accepted by other mainstream allied health care providers [29, 30]. This has included, among other strategies, a lack of clear definitions for key terms such as “chiropractor”, “diagnosis”, and “competency”, a lack of a whole-hearted acceptance of an evidence-based approach to standard educational development and clinical practice [29], and silence on known historically important traditional beliefs that have consequences for unwanted practitioner behaviours (*subluxation / vitalism*) [27, 31]. Also incorporated are a lack of transparency around CP inspections [53], negotiated settlements on accreditation standards with disparate and vocal interest groups, and seemingly placing the perceived specialness or preservation of chiropractic above the interests of the patient [25].

There was a reluctance to whole-heartedly embrace an evidence-based approach to accreditation standards. This was seen in a number of ways. Reservations were expressed about the possibility of overlooking clinical experience and teaching skills if CPs adopted the industry standard of PhD qualifications for academic staff. Also it was demonstrated by the CCE (USA) and CCE (Canada) expectations that chiropractic students should be able to understand and select methods for evaluating spinal articular subluxation or dysfunction [11] [13]). This is remarkable, as the term ‘*subluxation’* does not represent a reproducible clinically diagnosable entity [54]. Currently ‘*subluxation*’ remains a theoretical concept without evidence for its existence or ability to impact on the well-being of patients [55–57] and the intra and inter-reliability of students’ or practitioners’ ability to detect ‘*subluxations*’ is little better than chance [58–60].

Some of the CCE experts expressed the view that ‘traditional’ conservative and vitalist views are a personal belief and therefore outside the scope of the CCE standards and that any such regulation would be censorship of a CPs academic freedom [25]. It is true that academic freedom is “a system of complementary rights and obligations entitled to teachers and students as free enquirers” [61] and a fundamental right for CPs. However, academic freedom is held in tension with “academic duty” which requires sustained competency in pedagogy, graduate competency essentials, and accountability [62, 63]. By holding this tension, career competence and adherence to accreditation standards are supported [64]. Consequently, academic freedom that facilitates anti-vaccination views [19], the non-guideline use of X-rays [18], or beliefs that spinal manipulation is effective against AIDS [22], cannot be seen as supporting career competence and will need to be addressed in future revisions of educational standards.

CCE experts were aware that chiropractic education also faced many of the same challenges as other allied healthcare educators and these include that regulatory standards and processes should aim to be fair, transparent, and objective [65]. Also standards should make compliance straightforward and non-compliance easily measured and determined [66]. However, the reality is complex and a subject of concern for those engaged with educational standards and the accreditation processes [67–72]. It is agreed by accreditation researchers that there needs to be a common understanding of the language used by the stakeholders to accomplish accreditation and healthcare reform [73]. A lack of clarity results in an inability to gain a clear understanding of the issues at hand and fully engage all stakeholders to establish a shared agenda, establish goals, or methodologies for evaluating changes [73]. The issue of a lack of clarity is especially relevant for CCEs, when creating and reviewing accreditation standards and processes, as there is no widely agreed definition of chiropractic or its scope of practice. The absence of a common understanding has implications for the creation of an internationally homogeneous quality set of essential accreditation standards or measures of entry-level graduate competencies upon which CCEs can conduct reliable and valid assessments across all CPs. Prominent chiropractic researchers have called this an urgent task that must be undertaken for the profession to remain relevant in today’s evidence-based healthcare environment [74]. A method of adopting an evidence-based approach, where clinical uncertainties exist, has been proffered and warrants consideration for situations like this [75]. Medical education research has responded in a similar manner by seeking empirical, evidence-driven models of professional practice characteristics wherever possible [76]. Chiropractic education and accreditation should be no less diligent.

The interviews with CCE experts revealed that there are some positive signs that the journey for chiropractic from craft to profession is happening, albeit slowly. As an example, there are the beginnings of evidence of a general “soft” and “conditional” acceptance of the mainstream healthcare education standards of a scientific method, an evidence-based practice approach, and competency-based assessment of student learning outcomes [25]. Further, the definition of chiropractic appears to be moving slowly toward that of a practitioner who deals with musculoskeletal issues [30]. Also, CCEs appear open to the possibility of exploring innovative dimensions to accreditation such as the impact of student (and possibly staff) personality on clinical decision making [25, 77].

### Is there a way forward?

We argue that if the chiropractic profession is to become respectable, the “raison d’être” of CCEs should be to take a more upfront stand and better serve the patients’ best interests. There can be no dichotomous mindset that takes a compromised position in the face of known evidence, especially when it has implications for patient quality of care and safety. This can be accomplished by embracing and pursuing the scientific model and an evidence-based approach to clinical practice, in the same manner as other twenty-first century allied-health professions.

For CCEs to go down this path they will need to be sufficiently resourced. If, as recent studies suggests, there is a professional protective mindset that has a low tolerance to research scrutiny [53, 78], then these resources will need to be counterbalanced by the inclusion of a range of skilled people (educators, researchers, business managers, site inspection experts) from within and outside the profession, to develop and implement strategies to engage CPs on this journey. We have created a list of more specific recommendations and their justifications and these are seen in Table 1 in Additional file 1.

A pivotal and important question is “how do we judge the effectiveness of CCE accreditation standards and processes?” Some CCEs have taken the step to voluntarily seek accreditation with larger agencies, unfortunately others have not. Nonetheless, key indicators should be sought that demonstrate that the CCEs are continuing to improve the quality of chiropractic practitioners and that this is resulting in improvements of the quality of patient care. This may need to be at both an organisational and individual level. Early steps in this direction could be to move toward greater transparency. This involves, among other things, publishing CCE executives’ selection criteria such as qualifications and experience for the processes they undertake, the criteria and evidence used for deriving standards and procedures, how site inspection processes are reliable and valid, and the final report findings [53].

The move toward mainstream healthcare acceptance could be facilitated by improving links with medical education accrediting bodies (the ACGME [79] or CanMEDS [80]) who have vast experience and resources that CCEs may be able to learn from. Also they could explore links with other healthcare profession accreditation experts to join in public health initiatives and research, a strategy used by podiatrists in their similar move from craft to profession [81].

Finally, the CCE-International requires re-invigorating. All CCEs will need to be wholeheartedly committed to it. The intent is to have an overarching organisation that will drive the creation of professional definitions to underpin high quality equivalent international accreditation standards and processes. This will begin to address portability and respectability issues. The CCE-International could take the lead in exploring new and innovative dimensions of accreditation standards and processes and encouraging further integration into mainstream healthcare by encouraging the exploration of hospital placements for undergraduate student education.

## Conclusion

A primary concern is that all CCE organizations do not wholeheartedly embrace the standards of other mainstream allied-healthcare providers. Instead some appear to choose to continue on the same path, and not adopt contemporary values and standards, consequently it is our opinion that the chiropractic profession is likely to become increasingly left behind. This possibility raises the question of how much longer governments and health agencies will tolerate this inaction that allows graduates of accredited programs to support, from a non-evidence-based ‘philosophical’ perspective aberrant opinions such as anti-vaccination beliefs, the non-guideline use of diagnostic tests such as plain X-rays, and excessive non-indicated care, before imposing regulations. It should be borne in mind that regulations rarely are enacted to increase freedoms, by their very nature they tend to be restrictive. In the words of Michael Klepper “If we don’t make our own future, it will be made for us”, and we believe this resonates for the administration and oversight of chiropractic education [82].

## Supplementary information

**Additional file 1.** Table of Recommendations.

## Data Availability

Not applicable.

## Abbreviations

CCE: Council on chiropractic education
CP: Chiropractic program
