# Supplementary material for: The accreditation role of Councils on Chiropractic Education as part of the profession's journey from craft to allied health profession: a commentary
Source: Chiropr Man Therap. 2020 Jul 22;28:40. doi: 10.1186/s12998-020-00329-2 (PMC7374969; doi:10.1186/s12998-020-00329-2)
Supplement: Supplementary file 1 — Additional file 1. Table of Recommendations. [file 12998_2020_329_MOESM1_ESM.docx]

**Table 1: Table of Recommendations**

|  | **Recommendations** | **Justification** |
| --- | --- | --- |
| 1 | Internationally uniform definitions of basic terms such as chiropractic, diagnosis, and scope of practice are required. | Uniform and high quality methods of assessment for student learning-outcomes, and site inspection reports can be created to create standardised assessment of CPs across CCEs. Common standards would ensure and safeguard patient safety and care and support global workforce standardisation. |
| 2 | Creation of an internationally acceptable set of equivalent accreditation standards and graduate competencies. | For greater public confidence, graduate chiropractic homogeneity and workforce portability. |
| 3 | Reliable and valid measures for assessing student learning and CP performance. | Uniform assessment of CPs can create and allow for more accurate baseline measures from which quality improvements can be monitored. |
| 4 | CCEs adopt a wholehearted approach of science, EB practice and patient centred care to all aspects of standards and processes | Facilitate the integration into mainstream health care. |
| 5 | Funding sources be identified for CCEs. | This would allow CCEs to conduct their own quality improvements such as staff training and employ highly qualified people without a conflict of interest |
| 6 | CCEs should consider specialised further education for their executive members relevant to their roles, as well as specialist recruitment | This would provide CCEs with skill sets to manage the varied professional interest groups, establish standardised training for members and site inspections, develop strategies to increase CP compliance, and have a greater potential for promoting interdisciplinarity. |
| 7 | Facilitate research that explores an outcomes-based and prescriptive approach to the competency levels of graduating chiropractic students. | This will develop, inform and improve regulatory standards. |
| 8 | Move toward minimum faculty qualifications of a PhD. | This would improve the educational standing of chiropractic education and enhance research capability and quality. |
| 9 | Provide student hospital placements | Improve graduate student quality and interdisciplinarity skills. |
| 10 | Investigate innovative dimensions of student clinical decision making such as personality type. | Improve graduating students’ clinical decision making skills |
| 11 | Address unorthodox (vitalism and ‘subluxation’) practice patterns in CCE accreditation standards | Align chiropractic education with contemporary EB approaches to health profession education |
| 12 | CCEs should develop measures to monitor their own performance, both as an organisation and as individuals | Accountability would enhance the reputation of CCEs |
| 13 | CCEs should adopt a policy of transparency on all functions. | This is an industry wide trend and would enhance the reputation of CCEs. |
